# MED10 Increases Cisplatin Resistance by Promoting PTEN Ubiquitination of Hepatocellular Carcinoma

**DOI:** 10.2174/0115680096330893241221141235

**Published:** 2025-04-03

**Authors:** Qiucheng Cai, Jianyong Liu, Junyang Xiao, Jianwei Chen, Lizhi Lv, Fang Yang

**Affiliations:** 1 Department of Hepatobiliary Surgery Fuzhou Clinical Medical College of Fujian Medical University Fuzhou Fujian, 350025 China;; 2 Department of Hepatobiliary Surgery, 900 Hospital of The Joint Logistics Team, No. 156 The Second West Ring Road, Fuzhou, Fujian, 350025, People’s Republic of China, China

**Keywords:** Hepatocellular carcinoma, HCC, MED10, cisplatin resistance, PTEN, ubiquitination

## Abstract

**Objective:**

Hepatocellular carcinoma (HCC) is a highly prevalent malignant tumor, ranking as the third leading cause of cancer-related deaths worldwide. Despite advances in chemotherapy, many patients exhibit limited therapeutic efficacy, ultimately leading to cisplatin resistance. Thus, an in-depth investigation into the molecular mechanisms underlying cisplatin resistance is critically needed.

**Materials and Methods:**

This study utilized the GEPIA dataset to analyze MED10 expression and its association with HCC. MED10 expression levels in normal and HCC tissues were quantified *via* PCR and immunohistochemistry. HCC cell proliferation was assessed through cell viability and colony formation assays, while apoptosis rates were measured using flow cytometry. To examine PTEN ubiquitination, Western blot analysis was conducted *in vitro*. Additionally, xenograft tumor models were employed using BALB/c nude mice (male/female, 6 weeks old, 18-22 g) to evaluate cellular proliferation *in vivo*.

**Results:**

The findings reveal a pivotal role for MED10 in driving cisplatin resistance in HCC by promoting PTEN ubiquitination. MED10 expression correlated with HCC malignancy, and MED10 knockdown significantly reduced the IC_50_ of cisplatin in SMMC-7721, HepG2, and MHCC97-H cell lines. MED10 overexpression significantly decreased PTEN protein levels, which was reversed by the ubiquitination inhibitor TAK-243, while PTEN mRNA levels remained unaffected by MED10 overexpression or TAK-243. Both *in vitro* and *in vivo*, MED10 enhanced cisplatin resistance by promoting PTEN ubiquitination in HCC cells. These results offer valuable insights into the molecular mechanisms underlying MED10 expression and its role in cisplatin resistance in HCC.

**Conclusion:**

MED10 enhances cisplatin resistance by promoting PTEN ubiquitination in HCC cells.

## 1 INTRODUCTION

Hepatocellular carcinoma (HCC) remains a predominant malignancy, currently ranked as the third leading cause of cancer-related mortality worldwide, accounting for approximately 830,000 deaths annually [1]. In the past two decades, early-stage HCC diagnoses have increased markedly, with advancements in surgical resection and radiofrequency thermal ablation enhancing five-year survival rates to approximately 70% [2]. Nevertheless, around half of these patients experience relapse following surgery [3]. For those with intrahepatic or systemic metastasis, the prognosis is particularly grim due to aggressive tumor progression, limited therape- utic options, and poor outcomes, making curative treatment challenging [4, 5]. Consequently, an in-depth exploration of HCC’s molecular landscape is essential to identify robust prognostic markers and viable therapeutic targets.

Chemotherapy remains a principal intervention for patients with advanced-stage liver cancer [6, 7]. The recommended treatments for inoperable HCC include tyrosine kinase inhibitors and the combined administration of atezolizumab and bevacizumab. Although cisplatin is not widely used for HCC, its effects on this carcinoma type have been the subject of various studies. Despite prolonged chemotherapy regimens, a substantial proportion of patients exhibit reduced responsiveness and eventually develop cisplatin resistance, limiting treatment efficacy [8]. Therefore, continued investigation into the molecular mechanisms driving cisplatin resistance is imperative. Traditional pathways of chemoresistance have been extensively explored, particularly those involving genetic alterations in DNA damage repair (DDR) genes, increased detoxification enzyme activity, repressive chromatin configurations, and drug efflux mechanisms mediated by the overexpression of multidrug resistance-associated protein 2 [9]. In light of these challenges, compounded by the adverse prognosis and limited therapeutic success associated with persistent chemoresistance, a deeper examination of the molecular underpinnings specific to HCC is critically warranted.

Transcription plays a pivotal role in the cellular mechanisms governing protein synthesis in eukaryotic cells. Notably, cancer cells frequently exhibit elevated transcriptional activity, which supports their increased metabolic rates and rapid proliferation [10]. This phenomenon highlights the potential of targeting transcriptional processes in anticancer therapeutic strategies. The RNA polymerase II transcription mediator subunit 10 (MED10), a key component of the evolutionarily conserved Mediator complex in humans, is critically involved in this process. Existing literature emphasizes MED10's function as a mediator that connects regulatory proteins to RNA polymerase II (Pol II), thereby meticulously regulating Pol II-dependent transcriptional processes [11]. A growing body of evidence suggests that alterations in the expression of Mediator complex components significantly impact both tumor initiation and progression. However, these findings have been met with conflicting interpretations. In light of these discrepancies, the present study aims to explore the potential implications of dysregulated or aberrant MED10 expression in the pathogenesis of HCC. Additionally, the study seeks to elucidate the complex molecular mechanisms underlying this phenomenon.

This study examines the role of MED10 in promoting cisplatin resistance within the context of HCC. Initial analysis of the GEPIA (Gene Expression Profiling Interactive Analysis) dataset provides preliminary insights into the expression pattern of MED10. Building on this, the study undertakes a detailed exploration to uncover the molecular consequences and mechanisms through which MED10 mediates cisplatin resistance specifically in HCC. The findings of this research have the potential to offer valuable insights into the molecular pathways involving MED10, cisplatin resistance, and ubiquitination in the complex pathogenesis of HCC.

## 2 MATERIALS AND METHODS

### 2.1 GEPIA Analysis

The online database GEPIA (http://gepia.cancer-pku.cn/index.html) was utilized to validate genes exhibiting significant correlations within the context of HCC. GEPIA serves as an interactive web platform, housing a comprehensive dataset comprising 9,736 tumor samples and 8,587 normal samples from the TCGA and GTEx projects, all of which underwent rigorous RNA sequencing. The platform enabled the generation of survival curves based on gene expression data, employing the log-rank and Mantel-Cox tests across 31 distinct cancer types. To further investigate gene expression correlations, a detailed analysis was conducted using specific TCGA expression data, encompassing both tumor and normal tissue datasets. All the figures in Fig. **1** were generated from GEPIA website (http://gepia.cancer-pku.cn/index.html).

### 2.2 Sample Collection

A total of 38 normal liver tissues and 38 HCC tissues were collected from the 900 Hospital of The Joint Logistics Team (China). The inclusion criteria for the study were as follows: confirmed histological diagnosis of HCC, haven't received any antitumor therapy prior to surgery; and completion of the follow-up period. Metastatic HCC cases, treated cases before surgery, or with a follow-up time of less than 7 days were excluded from our study. All specimens were anonymized in accordance with ethical standards. Samples were collected immediately post-surgery and stored at -80°C. Ethical approval for this study was obtained from the Ethics Committee of the 900 Hospital of The Joint Logistics Team (No. 2023-FZ031), and written informed consent was acquired from each patient.

### 2.3 Histological Analysis

The HCC tissues were initially fixed in 4% paraformaldehyde for 1-2 days, followed by dehydration and paraffin embedding. Histological assessments were conducted using immunohistochemistry on 5μm-thick sections prepared in the sagittal plane. Detailed information on the primary and secondary antibodies employed in this study is provided in Supplementary Table **S1**.

### 2.4 qRT-PCR

For RNA extraction, cells or tissue samples were collected, PBS-washed, and homogenized using Trizol reagent (Life Technologies, USA). Following nuclear-cytoplasmic fractionation and homogenization, RNA isolation was performed according to the manufacturer's instructions. cDNA synthesis was carried out using the iScript cDNA Synthesis Kit (BIO-RAD, USA). Quantitative PCR analysis was conducted with the SsoFast EvaGreen Supermix system (BIO-RAD, USA), with all expression levels normalized to GAPDH. The primers utilized in this study are detailed in Supplementary Table **S2**.

### 2.5 Cell Culture and Stimulation

The WRL68 and HepG2 cell lines were purchased from ATCC (USA), while the QSG-7701, SMMC-7721, and MHCC-97H cell lines were sourced from the Institute of Cell Biology, Chinese Academy of Sciences (Shanghai, China). All cell cultures were maintained according to the manufacturer's guidelines and seeded onto T-75 flasks, which were incubated in a 5% CO_2_ atmosphere at 37°C, with medium changes every two days. To investigate the effects of the ubiquitination inhibitor TAK-243, MED10-overexpressing cells were treated with 10 nM TAK-243 (Active Biochem, San Jose, CA, USA) in 0.1% DMSO for 24 hours.

### 2.6 Western Blot Analysis

Total protein was extracted from cultured cells using RIPA buffer (Beyotime, China). Half of the cells were used for nuclear-cytoplasmic fractionation, and the remaining cells were processed for whole-cell analysis. After fractionation, specific marker proteins were analyzed by Western blot to confirm the purity of the fractions. GAPDH served as the marker for the nuclear fraction, while β-actin was used for the cytoplasmic fraction (no β-actin was detected in the nuclear fraction, and no Lamin B was detected in the cytoplasmic fraction). The quality and concentration of the extracted proteins were quantified using a BCA assay (Beyotime, China). The protein samples were subsequently diluted and incubated at 100°C for 5 minutes. Equal amounts of protein were loaded onto a 12% gel, obtained from the TGX Stain-Free FastCast Acrylamide Kit (Bio-Rad, USA), and subjected to electrophoresis. After electrophoresis, the proteins were transferred to a 0.45μm PVDF membrane (GE Healthcare, USA). The membranes were then blocked with non-fat dry milk (Beyotime, China) for 1 hour, followed by incubation with primary antibodies overnight at 4°C. Afterward, secondary antibodies were applied and incubated for 2 hours at room temperature. Following extensive washing, the membranes were visualized using a ChemiDoc MP Imaging System (Bio-Rad, USA), with chemiluminescent HRP substrate (Millipore, USA). GAPDH served as the reference control for cytoplasmic and whole-cell protein normalization, while Lamin B was used for the nuclear fraction. Detailed information regarding the specific primary and secondary antibodies used in this study is presented in Supplementary Table **S1**.

### 2.7 Cell Viability and Colony Formation Analysis

Cell viability was determined using the MTT assay (Abcam, UK), with results expressed as percentages relative to control samples subjected to identical conditions. Cell proliferation was quantified as a fold change in comparison to initial viable cell counts. Colony formation assays followed established protocols [12].

### 2.8 Immunofluorescence

For immunofluorescence analysis, cells were trypsinized, seeded onto chamber slides (Millipore, USA), and allowed to adhere for 24 hours. Fixation was performed with 4% paraformaldehyde, followed by permeabilization with 0.5% Triton-X. A blocking step with 2% bovine serum albumin was applied before staining. Cells were incubated with primary antibodies for 12–18 hours, followed by secondary antibody incubation for 1 hour, and counterstained with DAPI for 10 minutes. Fluorescence images were captured using a confocal microscope and analyzed with Image Pro PLUS software. Details on primary and secondary antibodies are provided in Supplementary Table **S1**.

### 2.9 Flow Cytometry

To evaluate apoptosis, trypsinized cells were washed and resuspended in PBS containing 2% FBS, then stained with Annexin V-PE (Beyotime, China) to assess apoptotic status. DAPI was added to each sample at least 1 minute before analysis to exclude non-viable cells. Flow cytometric analysis was conducted on a FACS Calibur system (BD, USA), with data processed using FlowJo v10 software (USA).

### 2.10 Lentivirus Transduction

Custom-designed lentiviruses containing MED10 siRNA or PTEN siRNA were generated by VectorBuilder (Santa Clara, USA), with construct sequences available upon request. Lentiviral transduction was performed in the cell culture medium following previously established protocols, and transduction efficiency was evaluated by assessing GFP fluorescence using a DMI3000B microscope (Leica, Germany).

### 2.11 Xenograft Tumor Animal Experiments

Thirty healthy BALB/c nude mice (half male/half female, 6 weeks old, 18-22 g) were purchased from the animal center of Fujian Medical University and housed in specific pathogen-free conditions (23-25 °C, 50-60% relative humidity and 12-hour light/dark cycle lighting ). Dry, clean, and well-ventilated cages with sufficient space were provided. Nutritionally balanced food and clean drinking water were supplied. All animals were housed in identical cages with the same environmental conditions, such as temperature, humidity, and lighting. Before the xenograft tumor cell implantation, all the mice were randomly assigned to five groups (N = 6) using a random number table. The experimenters were blinded to the treatment groups to minimize bias. Only the research designer was aware of the group allocation at the different stages of the experiment (during the allocation, the conduct of the experiment, the outcome assessment, and the data analysis). The order in which treatments were administered was randomly selected. For the experimental procedure, SMMC-7721 cells from designated groups were subcutaneously injected into the mice. The control mice (si-NC group) received an injection of negative control siRNA-treated SMMC-7721 cells and saline injection. Anaesthesia was administered intraperitoneally using ketamine (100 mg/kg body weight) and acepromazine (5 mg/kg body weight) to reduce pain. They were housed in groups to reduce distress. No adverse event was observed. The humane endpoints included: 1. tumor reaches a certain size (1500 mm^3^), causing pain and discomfort; 2. significant and/or rapid weight loss; 3. lethargy, hunched posture, labored breathing, or other visible signs of distress; 4. ulceration, necrosis, or other severe complications occurred in the tumor or surrounding tissues. Tumor volumes were measured every 5 days using the formula: volume (mm^3^) = (length × width^2^)/2. Tumor weights were recorded 25 days post-injection. Mice were sacrificed using cervical dislocation to ensure rapid euthanasia.

### 2.12 Statistical Analysis

Statistical analyses were conducted using SPSS 17.0 Software (SPSS, Inc., San Diego, CA, USA). Quantitative data were analyzed by one-way ANOVA, followed by Dunnett's multiple comparison post-hoc test to determine statistical significance. Data normality was assessed using the Shapiro-Wilk test; the equality of variances assumption was assessed using Bartlett's test. If the assumptions of normality and homogeneity of variances are not met, log transformation is applied to the data. Results are presented as mean ± standard deviation, with a significance threshold of P < 0.05.

## 3 RESULTS

### 3.1 Expression of MED10 is Associated with Malignancy in Patients with HCC

An in-depth analysis was conducted utilizing the RNA-seq data from the GEPIA database to inform our subsequent investigations. Among thirty-one tumor types examined, MED10 displayed a significant variation in expression specifically within HCC (16.19 in tumor *vs*. 9.8 in normal, Fig. **1A**). These observations were further validated through the analysis of 529 samples (369 cases and 160 controls). Notably, MED10 was most highly expressed in HCC tissues, with expression levels showing an upward trend corresponding to cancer progression (Figs. **1B**, **C**). Furthermore, elevated MED10 expression was associated with poorer prognosis in patients with HCC (Fig. **1D**).

To elucidate the expression patterns of MED10 in patients with HCC, immunostaining was employed to quantify both cytoplasmic and nuclear MED10 levels in a cohort of 38 normal and HCC tissues. The analysis revealed increased expression of both cytoplasmic and nuclear MED10 in HCC tissues relative to normal tissues, with expression intensity rising with HCC grade (Figs. **2A**-**D**). Additionally, higher MED10 expression was linked to an unfavorable prognosis in patients with HCC (Figs. **2E**-**F**). Together, these results position MED10 as a potential marker of high-grade HCC, indicating its pivotal role in the progression of this cancer.

### 3.2 MED10 is Required for the Growth of HCC Cells

A significant increase in MED10 expression was observed in high-grade HCC tissues compared to low-grade HCC tissues. Interestingly, MED10 expression was also quantifiable in most low-grade HCC samples, suggesting that MED10 may play a fundamental role within HCC tissues broadly. Consequently, a comparative analysis of MED10 expression was conducted across three HCC cell lines and two normal liver cell lines. Elevated MED10 protein and mRNA levels were consistently observed in the HCC cell lines—SMMC-7721, HepG2, and MHCC97-H—relative to normal liver cells (Figs. **3A**-**C**), implicating MED10’s role in malignancy promotion.

To further investigate MED10's function in HCC progression, MED10 was silenced in three HCC cell lines (Fig. **3D**). Functional analysis revealed that MED10 knockdown led to a marked reduction in cell viability and proliferation over the culture period (Figs. **3E**-**G**). Consistently, MED10-deficient HCC cells, when cultured at equal starting densities, showed a diminished capacity for colony formation after three weeks (Figure **3H**). Collectively, these results underscore MED10 as a critical driver of HCC cell growth.

### 3.3 MED10 Mediates Cisplatin Resistance in HCC Cells

The working hypothesis proposed that MED10 may contribute to the reduced sensitivity of HCC cells to cisplatin. To test this, MED10 knockdown HCC cell lines exposed to varying doses of cisplatin were examined. As shown in Figs. **4A**-**C**, cisplatin exhibited a dose-dependent inhibition of cell viability in HCC cells. The IC50 values for each cell line, with and without MED10 knockdown, were calculated and are presented in Figs. **4D**-**F**. Notably, MED10 knockdown significantly lowered the IC50 for cisplatin in SMMC-7721, HepG2, and MHCC97-H cell lines (P < 0.01 compared to control). To further elucidate MED10’s role in cisplatin resistance, an apoptosis assay was performed. After 24 hours of cisplatin treatment, apoptosis levels in HCC cell lines were significantly elevated (P < 0.01 compared to control), with a pronounced increase observed in MED10 knockdown cells (Figs. **4G**, **H**, P < 0.01 compared to cisplatin alone). Together, these results highlight MED10 as a promoter of cisplatin resistance in HCC cells.

### 3.4 PTEN Ubiquitination is the Downstream of MED10 in HCC Cells

Existing literature highlights the influence of ubiquitin ligases on the susceptibility of malignant tumor cells to cisplatin, primarily through the modulation of various substrates. To explore the mechanism by which MED10 mediates cisplatin resistance in HCC cells, our investigation focused on identifying downstream target proteins influenced by MED10. Notably, previous studies have emphasized the role of phosphatase and tensin homolog (PTEN) in HCC pathogenesis *in vitro* [13, 14]. Based on this, it was hypothesized that PTEN might be one of the target proteins regulated by MED10.

To determine whether MED10 modulates PTEN expression, PTEN levels in SMMC-7721 cell lines were analyzed after MED10 knockdown using Western blot. The results showed that MED10 knockdown led to an increase in PTEN expression *in vitro* (Fig. **5A**). mRNA analysis confirmed that MED10 knockdown effectively reduced MED10 mRNA levels (Fig. **5B**, P < 0.01 compared to si-NC) without affecting PTEN mRNA levels (Fig. **5B**). This study further assessed the protein levels of MED10 and PTEN in MED10-overexpressing HepG2 cells, with or without treatment with the ubiquitination inhibitor TAK-243 (Fig. **5C**). The data indicated that MED10 overexpression significantly reduced PTEN protein levels, a phenomenon that was reversed upon TAK-243 treatment. However, mRNA analysis of MED10 and PTEN indicated that neither MED10 overexpression nor TAK-243 treatment affected PTEN mRNA levels.

Immunofluorescence staining revealed that PTEN (shown in red) was predominantly localized in the nucleus, while MED10 (depicted in green) was present in both the nucleus and cytoplasm. This suggests that the interaction between PTEN and MED10 primarily occurs within the nucleus (Fig. **5E**). Additionally, co-immunoprecipitation experiments demonstrated a direct interaction between PTEN and MED10. Notably, MED10 knockdown resulted in a significant reduction in PTEN ubiquitination levels (Figs. **5F**-**H**). In summary, our findings suggest that PTEN functions as a downstream target of MED10, with this interaction playing a pivotal role in modulating cellular response to cisplatin. The regulation of PTEN by MED10 appears to occur through post-translational ubiquitination rather than changes in RNA transcription.

### 3.5 MED10 Increases Cisplatin Resistance by Promoting PTEN Ubiquitination of HCC In Vivo and In Vitro

To comprehensively elucidate the role of PTEN ubiquitination in cisplatin resistance within HCC cells, a series of experiments were conducted involving PTEN knockdown in SMMC-7721 cell lines. Subsequently, a range of functional assays were employed to investigate the impact of MED10 on cisplatin resistance *via* regulation of PTEN ubiquitination. These assays included apoptosis assays (Figs. **6A**, **B**), cell viability assays (Fig. **6C**), and cell clone formation assays (Figs. **6D**, **E**). Our findings revealed a marked decrease in cell viability and a concomitant increase in apoptosis following MED10 knockdown and cisplatin treatment. Notably, these effects on cisplatin resistance were reversed when both MED10 and PTEN were knocked down simultaneously. Additionally, *in vivo* xenograft tumor experiments further confirmed these results, showing that combined knockdown of MED10 and PTEN alleviated cisplatin resistance (Figs. **6F**, **G**). These experiments strongly support the notion that MED10 contributes to the enhancement of cisplatin resistance in HCC cells, both *in vitro* and *in vivo*, by facilitating PTEN ubiquitination.

## 4 DISCUSSION

HCC is the most common form of primary liver cancer and represents a significant medical challenge. In 2012 alone, 782,000 cases of HCC were diagnosed, resulting in 746,000 deaths. The age-adjusted global incidence rate stands at 10.1 cases per 100,000 person-years, making HCC the sixth most prevalent cancer and the third leading cause of cancer-related mortality worldwide [15,16]. Notably, HCC has become a major cause of death among patients with cirrhosis, and its incidence is expected to rise in the coming years. Despite advancements in treatment options such as surgical resection, ablation, and liver transplantation, the prognosis for patients with HCC remains poor due to high rates of metastasis and recurrence [17-19]. Thus, identifying early biomarkers and effective therapeutic targets for HCC is of critical importance. In this regard, our analysis of the GEPIA dataset initially revealed a correlation between MED10 expression and malignancy in patients with HCC. Furthermore, MED10 plays a critical role in promoting the growth of HCC cells. These findings not only contribute to the growing body of HCC research but also offer potential insights for future investigations in this area.

Cisplatin (cis-diamminedichloroplatinum II), a widely used chemotherapeutic agent, has demonstrated substantial efficacy in the treatment of various cancers, including those affecting the testis, ovary, head, neck, and lung. In particular, cisplatin, often combined with etoposide and bleomycin, has been highly effective in treating testicular cancer, with cure rates exceeding 95%. However, despite its success in testicular cancer, its efficacy against other malignancies is frequently hindered by intrinsic or acquired resistance. Overcoming cisplatin resistance remains a central challenge in cancer therapy. This study uncovered the role of MED10 in mediating cisplatin resistance in HCC cells. Additionally, MED10 may enhance cisplatin resistance by facilitating PTEN ubiquitination in both HCC cells and xenograft tumor models. These insights highlight the complex mechanisms contributing to cisplatin resistance and emphasize the need for novel strategies to overcome this obstacle in cancer treatment.

The precise orchestration of transcription, spanning both temporal and spatial dimensions, is crucial during organogenesis and is primarily governed by transcriptional activators and repressors. Disruptions to this finely tuned regulation can lead to physiological abnormalities and pathological conditions. The multiprotein MED complex functions as a bridge, facilitating the transmission of regulatory signals between factors and the basal transcription machinery. While some MED subunits, such as Trap80 and Med6, are essential for general gene transcription, others—like MED10, MED12, and MED13—are not universally required but play critical roles in the regulation of specific gene programs [20-22]. Despite the known involvement of MED in various cellular processes, its role in tumor development remains poorly understood. For instance, Zhao *et al*. reported elevated MED15 expression in breast cancer tissues, which was associated with increased TGFβ/Smad3 signaling. Inhibition of MED15 was shown to reduce metastatic behavior in a highly aggressive breast cancer cell line [23]. Additionally, decreased MED15 expression was observed in uterine leiomyosarcomas, independent of mutational status, and MED15 was proposed as a tumor suppressor in oral and oropharyngeal cancers [24, 25]. Conversely, reduced expression of MED1 has been linked to metastasis-related gene downregulation in melanoma, lung, and bladder cancers, while augmented MED1 activity was observed in prostate and breast cancers, where it functions as a key hub for nuclear hormone receptors [26-30]. In this study, analysis of the GEPIA dataset reveals elevated MED10 expression that correlates with malignancy in patients with HCC. Moreover, this study demonstrates the indispensable role of MED10 in HCC cell proliferation and progression. Cellular experiments and xenograft tumor models further reveal that MED10 enhances cisplatin resistance by promoting PTEN ubiquitination in HCC, both *in vitro* and *in vivo*. Consequently, MED10 emerges as a potential biomarker for predicting cisplatin resistance in the treatment of HCC.

The ubiquitin-proteasome system, essential for maintaining protein homeostasis and proper cellular function, regulates the degradation of most proteins, influencing biological processes at multiple levels, including stress response, DNA damage repair, and cell proliferation [31]. Disruptions in this system have been implicated in numerous diseases, including various cancers [32]. Ubiquitination, a multi-step process involving E1, E2, and E3 enzymes, governs substrate specificity and mediates the degradation of specific oncoproteins. Consequently, E3 ligases have emerged as promising therapeutic targets for anticancer interventions [33]. In liver cancer pathogenesis, ubiquitination plays a critical role, yet research into its involvement in liver cancer chemoresistance and the underlying mechanisms remains limited. Our study reveals that MED10 enhances cisplatin resistance in HCC cells and HCC xenograft tumor models by promoting PTEN ubiquitination. This underscores the pivotal role of ubiquitination, particularly PTEN ubiquitination, in chemoresistance within HCC.

PTEN, a well-known tumor suppressor in many cancers, is critical for regulating tumorigenesis and cellular self-renewal [34-36]. Molecular alterations in PTEN expression or activity are associated with a variety of cellular defects, contributing to carcinogenesis in both hepatic and non-hepatic cells. PTEN influences the activation of the PI3K and MAPK pathways through IGF-1/2, essential for hepatocyte proliferation. Additionally, a reciprocal regulatory interaction between PTEN and IGF-2 has been observed in various cancer cell types, including hepatoma cells [37-40]. PTEN's role in epithelial-mesenchymal transition (EMT), enhancing the migratory and invasive capacities of cancer cells, as well as its involvement in chromosomal stability and DNA repair *via* nuclear expression, further emphasizes its functional significance [41-43]. Although further research is needed to establish PTEN as a reliable, standalone prognostic marker for HCC, existing evidence strongly supports that mutations or dysregulated PTEN expression or activity are major drivers of unchecked hepatocyte proliferation and malignancy. Our findings add to this body of knowledge by suggesting that PTEN ubiquitination, potentially regulated by MED10, contributes to the promotion of cisplatin resistance in HCC cells. These cumulative observations reinforce PTEN’s integral role in the complex pathogenesis of HCC.

The limitations of the present study may include the following. First of all, our study may have inherent biases in the selection of cell lines or experimental conditions, which could impact the generalizability of the results. Secondly, While cell experiments and animal models provide valuable insights, there may be limitations in extrapolating the results to human patients due to differences in biology and disease progression between species. Also, the study may have limitations in sample size, both *in vitro* and *in vivo*, which could impact the statistical power and generalizability of the results.

## CONCLUSION

In conclusion, our study highlights MED10’s pivotal role in cisplatin resistance in HCC by promoting PTEN ubiquitination. A significant correlation between MED10 expression and malignancy was observed in patients with HCC, further demonstrating its essential role in the proliferation of HCC cells. Additionally, MED10 was found to enhance cisplatin resistance through PTEN ubiquitination, both *in vitro* and *in vivo*. These findings provide new insights into the molecular mechanisms by which MED10 mediates cisplatin resistance, offering potential therapeutic avenues for overcoming resistance in HCC treatment. It is essential to acknowledge the potential sources of bias, limitations of the animal model, and imprecision associated with the results. Future research should address these limitations to further validate and refine the understanding of the molecular mechanisms involved in cisplatin resistance in HCC.

## Figures and Tables

**Fig. (1):**
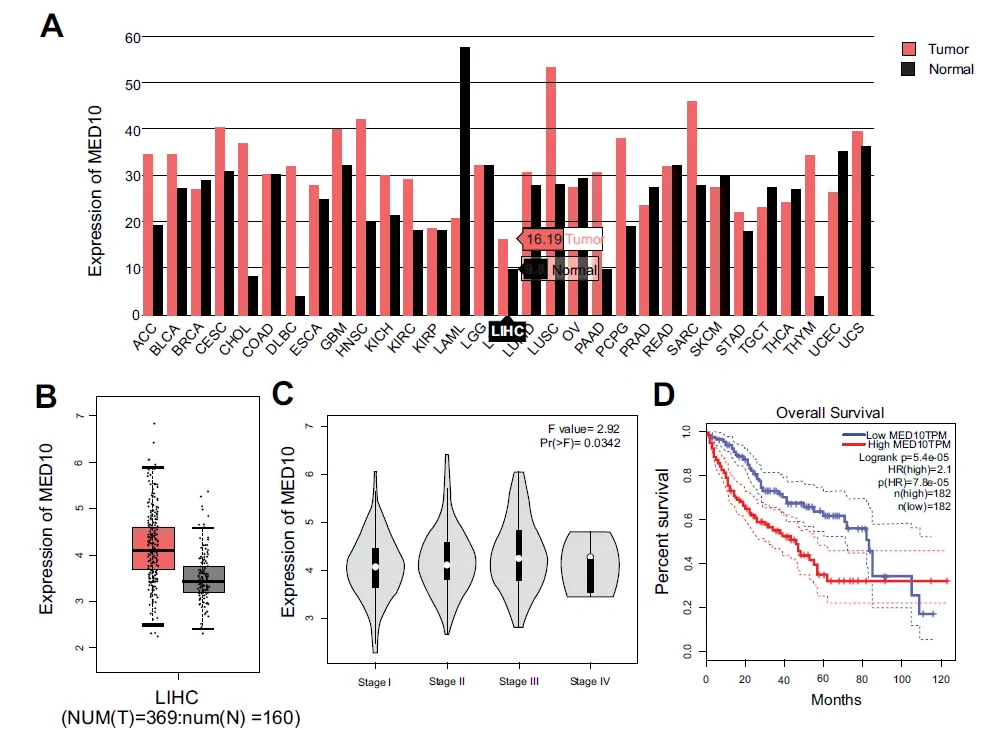
Expression of MED10 from GEPIA dataset. (**A**) The expression of MED10 in 31 types of cancers from the GEPIA dataset. (**B**) Comparison of MED10 expression in tumor samples (n = 369) and normal samples (n = 160). (**C**) MED10 expression across different cancer stages. (**D**) Overall survival analysis of patients with low or high MED10 expression.

**Fig. (2):**
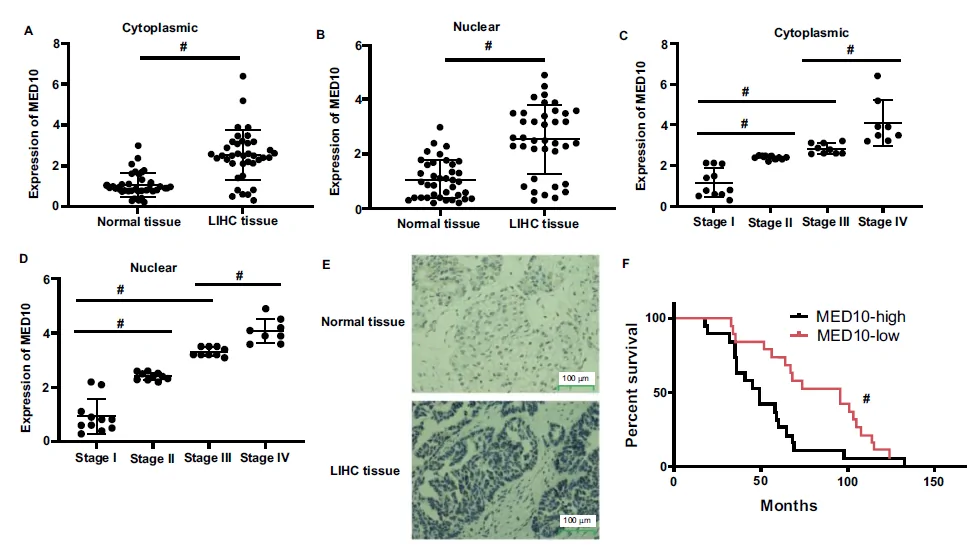
Expression of MED10 is associated with malignancy in patients with HCC. (**A**-**B**) Expression of MED10 in normal liver tissues and HCC tissues (n = 38). GAPDH was used for normalization. (**C**-**D**) MED10 expression across different stages of HCC (n = 38). GAPDH was used for normalization. (**E**) Immunohistochemical analysis of MED10 expression in normal tissue samples and HCC tissue samples. Scale bar = 100 μm. (**F**) Overall survival analysis of patients with low or high MED10 expression (n = 38). Error bars represent the means ± SEM of three independent experiments. #p < 0.05 between indicated groups.

**Fig. (3):**
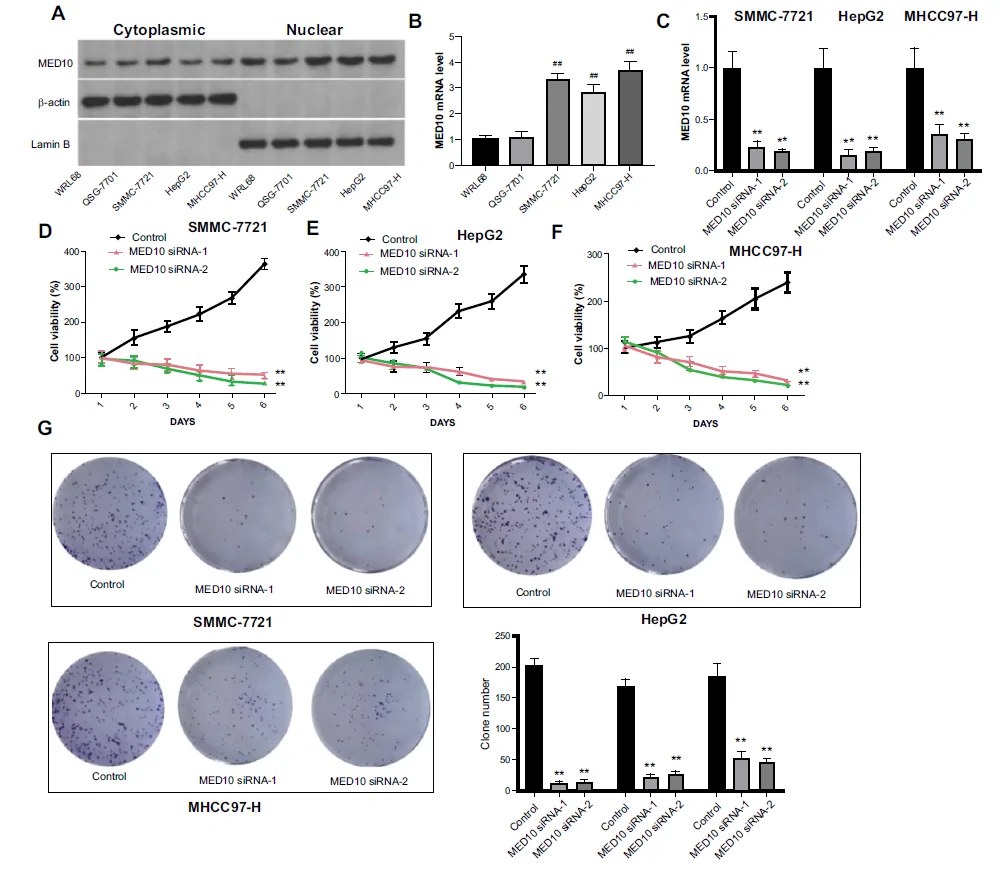
MED10 is required for the growth of HCC cells. (**A**-**C**) Protein localization and mRNA levels of MED10 in normal liver cell lines and HCC cell lines (n = 6). (**D**) mRNA levels of MED10 in HCC cell lines after MED10 knockdown (n = 6). (**E**-**H**) Cell viability and colony formation assays in normal HCC cell lines or MED10 siRNA-treated HCC cell lines (n = 6). (**E**-**G**) Cell viability assay (n = 6). (**H**) Colony formation assay (n = 6). Error bars represent the means ± SEM of three independent experiments. ∗∗p < 0.01 compared to Control, ##p < 0.01 compared to WRL68 cells.

**Fig. (4):**
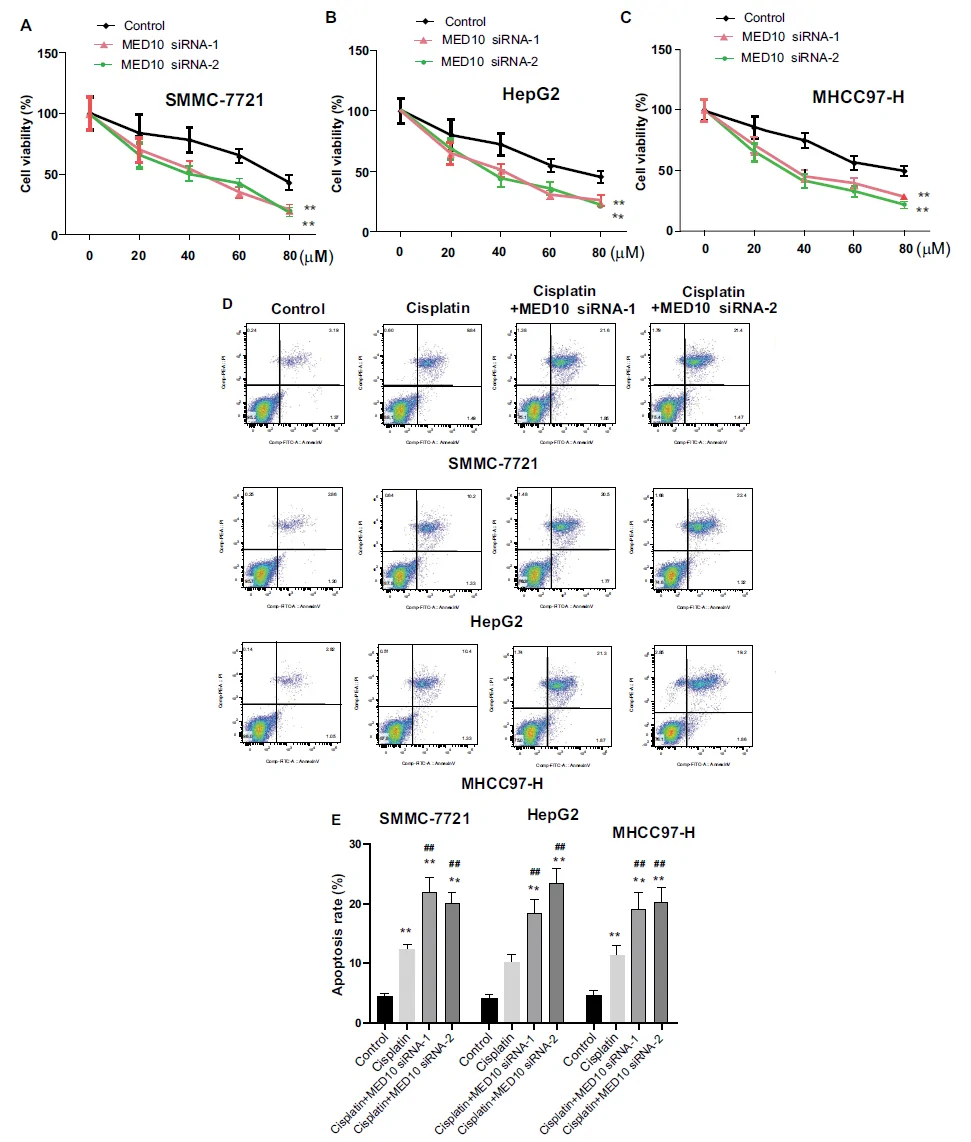
MED10 mediates cisplatin resistance in HCC cells. (**A**-**C**) Cell viability of normal HCC cell lines or MED10 siRNA-treated HCC cell lines after cisplatin treatment (n = 6). (**D**-**F**) The IC50 values of cisplatin in normal HCC cell lines or MED10 siRNA-treated HCC cell lines (n = 6). (**G**, **H**) Apoptosis in normal HCC cell lines or MED10 siRNA-treated HCC cell lines after cisplatin treatment (n = 6). Error bars represent the means ± SEM of three independent experiments. ∗∗p < 0.01 compared to Control, ##p < 0.01 compared to Cisplatin-treated group.

**Fig. (5):**
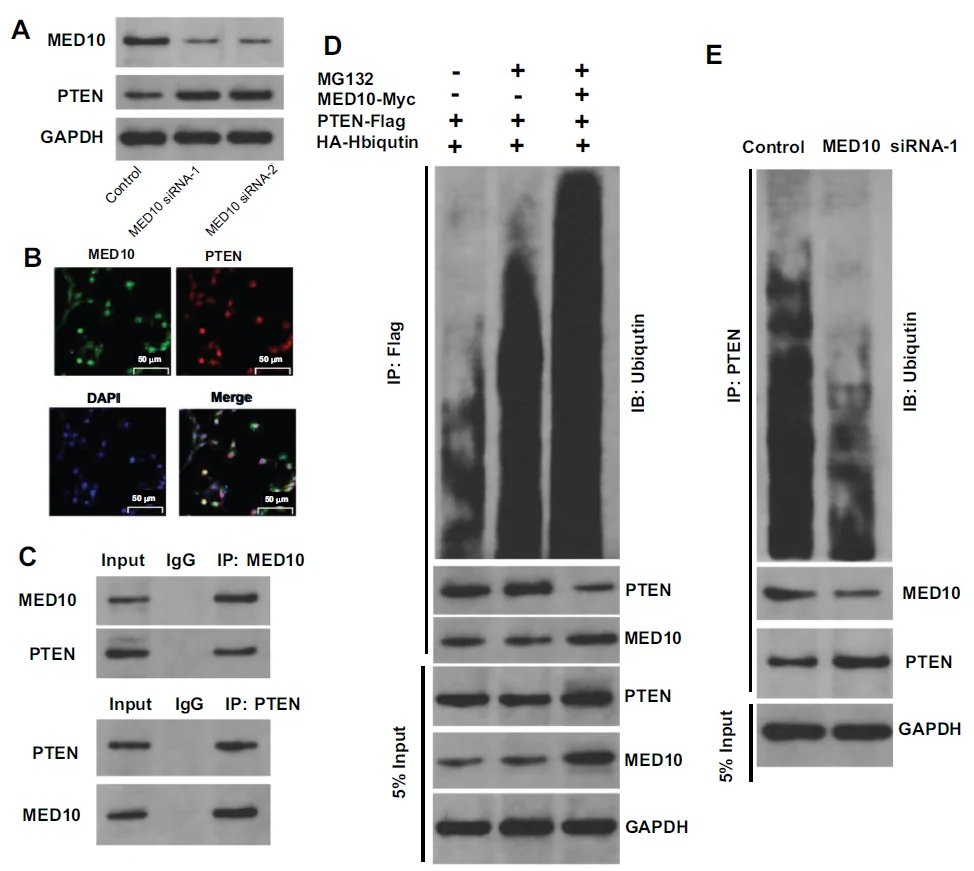
PTEN ubiquitination is downstream of MED10 in HCC cells. (**A**) Protein levels of MED10 and PTEN in HCC cell lines after MED10 knockdown (n = 6). GAPDH was used for normalization. (**B**) mRNA levels of MED10 and PTEN in HCC cell lines after MED10 knockdown (n = 6). (**C**) Protein levels of MED10 and PTEN in HCC cell lines after MED10 overexpression and TAK-243 treatment (n = 6). (**D**) mRNA levels of MED10 and PTEN in HCC cell lines after MED10 overexpression and TAK-243 treatment (n = 6). (**E**) Co-localization of MED10 and PTEN in HCC cells (n = 6). Scale bar = 50 μm. (**F**) Immunoprecipitation using anti-IgG, anti-PTEN, or anti-MED10 antibodies followed by western blot analysis of PTEN and MED10 in HCC cell lines (n = 6). (**G**) 293T cells were transfected and treated with/without MED10, then immunoprecipitated with anti-FLAG antibodies and analyzed by western blot using the indicated antibodies (n = 6). (**H**) MED10-silenced or control HCC cell lines were immunoprecipitated with anti-PTEN antibodies and analyzed by western blot using the indicated antibodies (n = 6). Error bars represent the means ± SEM of three independent experiments. ∗∗p < 0.01 compared to si-NC; ##p < 0.01 compared to OE-NC.

**Fig. (6):**
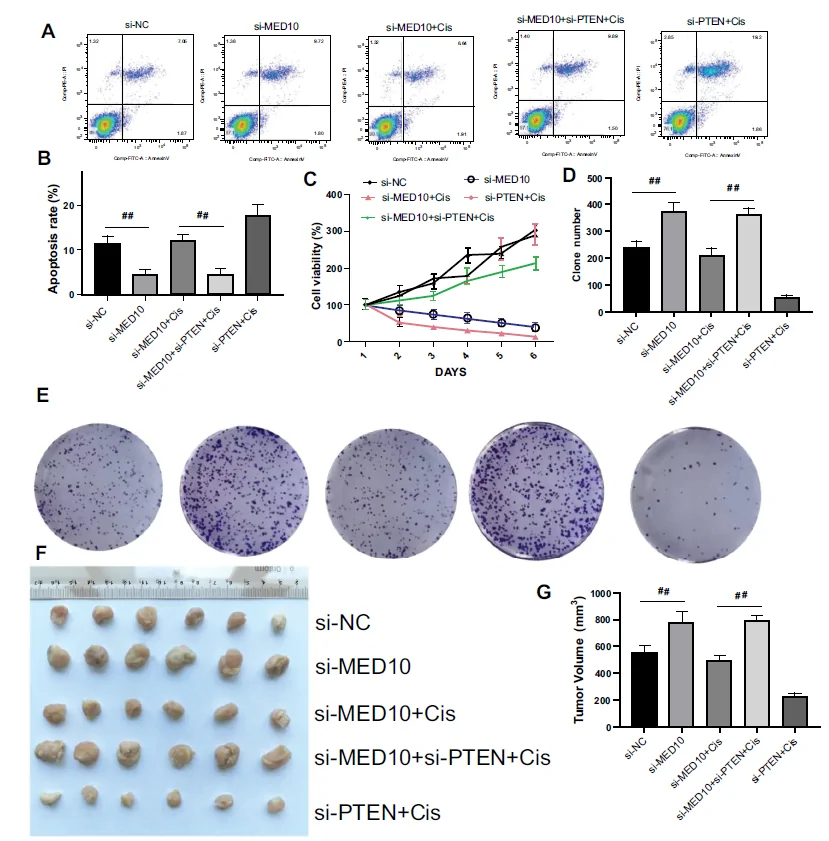
MED10 increases cisplatin resistance by promoting PTEN ubiquitination in HCC *in vivo* and *in vitro*. (**A**, **B**) Apoptosis in normal HCC cell lines or MED10, PTEN siRNA-treated HCC cell lines after cisplatin treatment (n = 6). (**C**) Cell viability in normal HCC cell lines or MED10, PTEN siRNA-treated HCC cell lines after cisplatin treatment (n = 6). (**D**, **E**) Colony formation assay in normal HCC cell lines or MED10, PTEN siRNA-treated HCC cell lines after cisplatin treatment (n = 6). (**F**, **G**) Xenograft tumor size in the control group or MED10, PTEN siRNA groups after cisplatin treatment (n = 6). Error bars represent the means ± SEM of three independent experiments. ##p < 0.01 between indicated groups.

## Data Availability

The data and supportive information is available within the article.

## LIST OF ABBREVIATIONS

DDR: DNA Damage Repair
GEPIA: Gene Expression Profiling Interactive Analysis
HCC: Hepatocellular carcinoma
MED10: Mediator Subunit 10
Pol II: Polymerase II

## STANDARD OF REPORTING

STROBE guidelines were followed.
