# Supplementary material for: MED10 Increases Cisplatin Resistance by Promoting PTEN Ubiquitination of Hepatocellular Carcinoma
Source: Curr Cancer Drug Targets. 2025 Apr 3;26(7):714–26. doi: 10.2174/0115680096330893241221141235 (PMC13678609; doi:10.2174/0115680096330893241221141235)
Supplement: Supplementary file 1 [file CCDT-26-7-714_SD1.pdf]

Supplementary Material

MED10 Increases Cisplatin Resistance by Promoting PTEN Ubiquitination of Hepatocellular Carcinoma

Qiucheng Cai[1,2], Jianyong Liu[1], Junyang Xiao[2], Jianwei Chen[1], Lizhi Lv[1,2] and Fang Yang[1,2,*]

[1]Department of Hepatobiliary Surgery, 900 Hospital of The Joint Logistics Team, No. 156 The Second West Ring Road, Fuzhou, Fujian, 350025, People's Republic of China; [2]Department of Hepatobiliary Surgery Fuzhou Clinical Medical College of Fujian Medical University Fuzhou Fujian, 350025 China

Supplementary Table S1. Antibodies used in the study.

Reagent	Company	Cat. No
Western blot analysis		
Primary antibodies		
Anti-MED10 antibody, rabbit polyclonal	ThermoFisher	PA5-111163
Anti-PTEN antibody, rabbit monoclonal	Abcam	ab267787
Anti-GAPDH antibody, rabbit polyclonal	Abcam	ab9485
Secondary antibodies		
Goat Anti-Rabbit IgG H&L (HRP)	Abcam	ab97051
Histological analysis		
Anti-MED10 antibody, rabbit polyclonal	ThermoFisher	PA5-111163
Goat Anti-Rabbit IgG H&L (HRP)	Abcam	ab97051
Innumofluorescence		
Anti-MED10 antibody, rabbit polyclonal	ThermoFisher	PA5-111163
Goat Anti-Rabbit IgG H&L (Alexa Fluor® 594)	Abcam	ab150080

Supplementary Table S2. Primers table.

**RT-PCR Source Identifier**		
**Primers**		
MED10	GGAAAGAACACCCGGAAATGA	GTGCTGCAACTGGGTAAACAC
GAPDH	AGTGTGACGTGGACATCCGCA	ATCCACATCTGCTGGAAGGTGGAC
